# Ecologically robust gut environment associated with personalized metabolic responses in a Japanese cohort

**DOI:** 10.1080/29933935.2025.2574930

**Published:** 2025-11-16

**Authors:** Chiharu Ishii, Miyuki Suzuki, Shinnosuke Murakami, Isaiah Song, Yoshiomi Soejima, Morimasa Kato, Shinji Fukuda

**Affiliations:** a Institute for Advanced Biosciences, Keio University, Tsuruoka, Yamagata, Japan; b Systems Biology Program, Graduate School of Media and Governance, Keio University, Fujisawa, Kanagawa, Japan; c Shimokitazawa Hospital, Setagaya, Tokyo, Japan; d ROHTO Pharmaceutical Co., Ltd, Ikuno, Osaka, Japan; e Yamagata Prefectural Yonezawa University of Nutrition Sciences, Yonezawa, Yamagata, Japan; f Innovative Microbiome Therapy Research Center, Juntendo University Graduate School of Medicine, Bunkyo-ku, Tokyo, Japan; g Gut Environmental Design Group, Kanagawa Institute of Industrial Science and Technology, Kawasaki, Kanagawa, Japan; h Transborder Medical Research Center, University of Tsukuba, Tsukuba, Ibaraki, Japan; i Metagen, Inc., Tsuruoka, Yamagata, Japan

**Keywords:** Fecal metabolome, gut microbiome, diet, robustness, personalized intestinal environment, machine learning, metabologenomics

## Abstract

The gut microbiota produces numerous metabolites that affect host physiology. However, the effects of daily diet on human fecal metabolome profiles and their robustness are not well understood, and examinations of intra-individual stability over multiple time points are limited. Here, we investigated the robustness of the human intestinal environment through fecal metabolome and microbiome profiling in response to daily dietary fluctuations. We analyzed 176 fecal samples from 25 healthy Japanese individuals subjected to three dietary regimens, including heterogeneous and homogeneous diets. Fecal metabolome and microbiome profiles were unique to each individual. Further in-depth analyses of seven of these individuals showed that these profiles were stable despite daily dietary fluctuations in six individuals. In addition, random forest classification successfully predicted each subject’s identity based on their metabolome profile. The correlation analysis also revealed that the food-metabolite and food-microbiome relationships were highly personalized. The findings from this study suggest that individual diet prior to sample collection is unlikely to influence the fecal metabolome and microbiome data to an extent that is not representative of the individual’s “normal” condition, which may lower barriers to future research on the gut environment and its implications for host health.

## Introduction

The gut microbiota and the metabolites they synthesize are strongly associated with host physiology. Both the gut microbiota structure and the metabolites produced by the gut microbiota are involved in disease development. For example, gut microbiome-derived metabolites such as deoxycholate, trimethylamine *N*-oxide, phenylacetyl glutamine, imidazole propionate, and 4-ethylphenylsulfate are associated with the development of hepatic cancer,^1^ atherosclerosis,^2-4^ cardiac disease,^5^ diabetes,^6^ and neurodevelopmental disorders,^7^^,^^8^ respectively.

Diet is one of the most important factors that impact both the gut microbiome and metabolome profiles. Differences in long-term dietary habits, such as between omnivores and vegetarians, alter short-chain fatty acid (SCFA) levels in humans.^9^ According to a previous analysis of short-term dietary perturbations, a dietary shift from a plant-based to an animal-based diet quickly altered the gut microbiome and SCFA levels.^10^ However, such a large shift in diet is not typical in daily life. The impact of daily dietary fluctuations on the fecal metabolome profile is not yet clear. In addition, various studies have compared fecal metabolome profiles between healthy individuals and patients, among people receiving dietary interventions, and among those exposed to metals and/or antibiotics.^11^ However, most of these studies that investigated the human fecal metabolome examined single time points from every individual for the purpose of intergroup comparisons, and studies examining intra-individual stability using multiple time points have been quite limited. The lack of basic information on the relationship between daily dietary variability and fecal metabolome profiles is a potential barrier to the use of metabolome profiles in gut health research, including applications such as health checkups and early detection of disease.

One study in the USA investigated the gut microbial metagenome and food records across 17 d, revealing a highly personalized correlation between bacteria and host food choices. However, the fecal metabolome profiles were not examined in this study.^12^ Among the few studies that have carried out longitudinal fecal metabolome analysis in individuals, a study of a 7-d homogeneous diet (consistent consumption of the same foods) period in an American cohort highlighted that homogeneous dietary conditions help to identify factors shaping the composition of the gut microbiota and its metabolic output.^13^ In addition, a recent study has shown that fecal metabolites exhibit relatively high stability during a one-month period when subjects consume their usual diet.^14^ However, not enough research has been conducted to compare the degree of stability within an individual who consumes a homogeneous diet or heterogeneous diet (varied meals at different time points). Therefore, it is difficult to clarify whether fluctuations in the intestinal environment are associated with diet. In addition, as people in different geographical regions have different eating habits and gut environments,^15^ studies of individuals in environments other than Western countries are needed.

We hypothesize that the human intestinal microbiome and metabolome exhibit unique, individualized profiles that remain relatively stable despite daily dietary fluctuations. To validate this hypothesis, we collected continuous time course data on the gut microbiome, fecal metabolome, and dietary intake from 25 healthy Japanese subjects across three dietary phases, including both heterogeneous and homogeneous diets, to investigate the robustness of the human intestinal environment in response to daily dietary fluctuations. Here, we show that the stability of the fecal metabolome and microbiome profiles in healthy humans does not change between heterogeneous and homogeneous diet shifts in most cases and that intestinal environmental factors are unique to each individual. In addition, the characteristics of individual metabolome profiles were robust, achieving over 92% accuracy in the identification of source donors by using a random forest algorithm. Our findings emphasize that not only human gut microbiome profiles, but also metabolome profiles have unique features in individuals regardless of daily dietary fluctuations. This finding suggests that it is possible to obtain individual-specific gut environment profiles without necessarily considering immediate dietary intake, which may contribute to the development of new approaches for health diagnostics and early disease detection using the gut environment.

## Results

### Daily dietary composition of healthy Japanese subjects in this study

We obtained gut microbiome, metabolome, and food information from 25 healthy Japanese individuals over three dietary phases. Clinical information about the subjects is shown in Table S1, and the experimental scheme of this study is shown in Figure S1A. During the first phase (Term 1), there were no dietary restrictions, but the consumption of medicinal drugs and dietary supplements was prohibited. The purpose of this phase was to profile the subjects' baseline dietary habits and gut environment. During the second phase (Term 2), the subjects ate only the meals that were provided to them. The menu for each meal was different but was consistent across all individuals. This phase was conducted to observe whether the similarity of profiles between individuals would change when they all followed the same diet. For the last phase (Term 3), the subjects again ate only meals that were provided to them, but during this phase, they consumed the same meal 15 times. The final phase was set to clarify whether the stability of fecal metabolome and gut microbiome profiles within individuals would change if they consumed a uniform diet for every meal. The number and frequency of fecal samples obtained during that period are shown in Figure S1B. During Term 1, the subjects recorded their meals by using provided forms and/or photographs. Commercially available boxed meals, frozen foods, fruits, sandwiches, rice balls, and foods in retort packaging were provided during Terms 2 and 3. The meals eaten by the subjects during Term 3 for 5 d are shown in Figure S1B, and the nutritional composition of the meals during Terms 1 to 3 are shown in Table S2.

**Figure 1.**
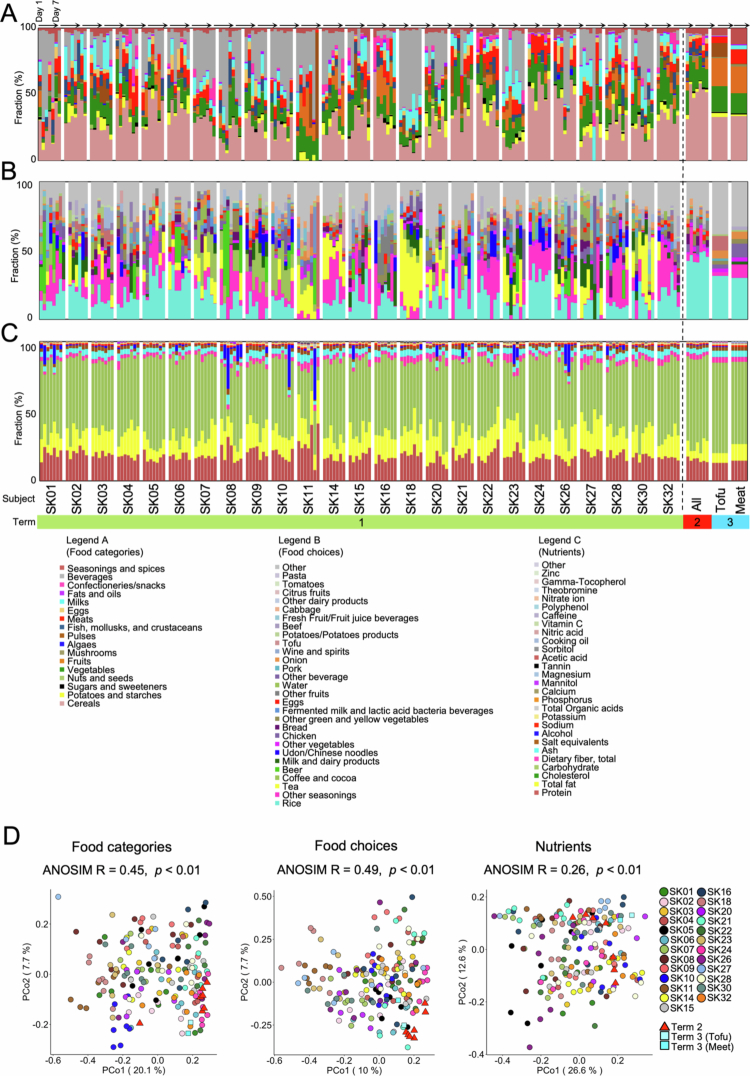
Daily dietary intake was individualized during the first term of the study. (A) Bar graph showing the fraction of food categories in the dataset for each subject, by day, over the study time course. All dietary data were collapsed into 17 food groups using the National Health and Nutrition Survey. (B) Fraction of the 28 most abundant food choices, by day, over the study time course. Low-abundance foods are grouped together as “Other”. (C) Fraction of the 27 most abundant nutrient intake shown according to the grams of each nutrient consumed per subject. Low-abundance nutrients are grouped together as ‘‘Other’’. (D) PCoA of food categories, food choices, and nutrient profiles based on Bray–Curtis dissimilarity with ANOSIM, comparing the profiles between subjects and terms.

The food categories, food choices, and nutrients that the subjects consumed are shown in Figure 1A–C. Principal coordinate analysis (PCoA) based on Bray‒Curtis dissimilarity and analysis of similarities (ANOSIM) using this dataset were conducted to compare individual food intake (Figure 1D). According to the ANOSIM results, broad food categories and specific food choices both varied widely among individuals. However, the nutrient intake was more consistent. In addition, the meals served during Term 2 and Term 3 did not deviate from the usual Japanese diet, as represented by the study's participants, in terms of nutrient composition.

### Gut microbiome profiles were unique among individuals

**Figure 2.**
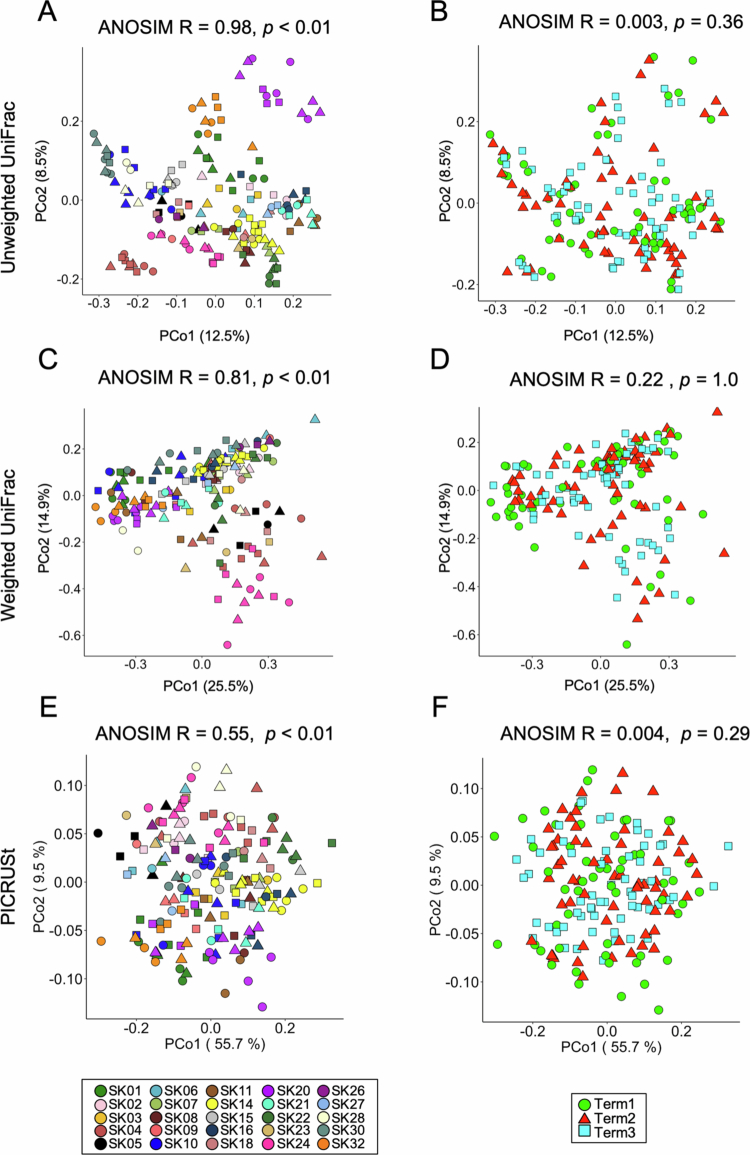
Gut microbiome profiles are clustered according to individuals rather than to dietary terms. (A–F) PCoA plots based on (A and B) unweighted UniFrac distances, (C and D) weighted UniFrac distances, and (E and F) Bray‒Curtis dissimilarity, with an ANOSIM used to compare the (A–D) intestinal microbiome profiles or (E and F) PICRUSt-predicted functional metagenome profiles among (A, C, and E) subjects or (B, D, and F) dietary terms.

To evaluate the impact of daily dietary fluctuations on gut microbial profiles, 16S rRNA-encoding genes were sequenced using MiSeq. A total of 3,099,385 16S rRNA gene sequence reads that passed a quality filter were assigned to 478 bacterial species. The genus-level microbial community structures and functional metagenome profiles predicted by Phylogenetic Investigation of Communities by Reconstruction of Unobserved States (PICRUSt) are shown in Figures S2 and S3, respectively. Unweighted and weighted UniFrac PCoA were performed to compare the microbial composition and abundance, respectively, and ANOSIM was used to evaluate the similarity between samples (Figure 2A–D). UniFrac PCoA and ANOSIM indicated that the profiles diverged depending on the individual but not on the dietary condition in both unweighted and weighted UniFrac analyses. Thus, the human gut microbial profiles (microbial composition and abundance) were unique to individuals, which is consistent with the results of previous studies.^16^^,^^17^ PCoA plots and ANOSIM of predicted metagenome profiles showed that the functional metagenome profiles were also more similar across individuals than across dietary conditions (Figure 2E, F). The ANOSIM score indicated that bacterial community structure was more varied between individuals in contrast to metagenome profiles, which is consistent with the idea of a core metagenome.^18^

### Fecal metabolome profiles were also unique among individuals

**Figure 3.**
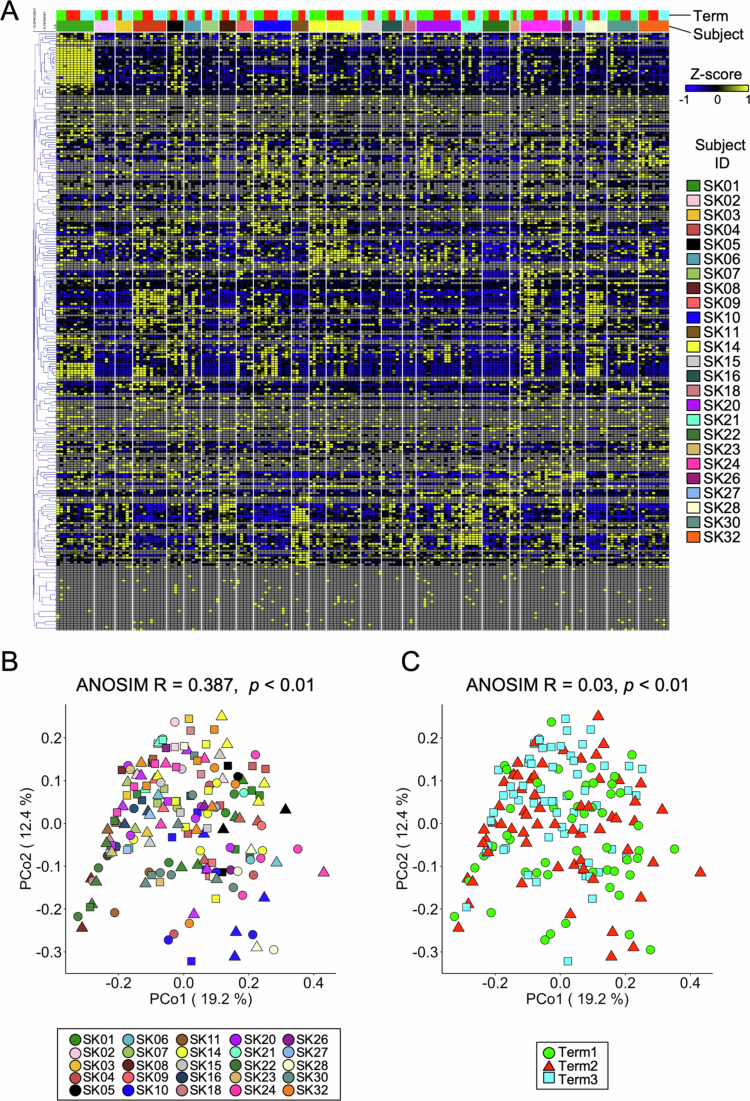
Fecal metabolome profiles are clustered according to individuals rather than to dietary terms. (A) Heatmap showing the concentrations of quantified metabolites, which were converted to Z-scores and displayed using a blue–yellow scheme. Gray indicates concentrations that were below the limit of detection. (B and C) PCoA plots of the fecal metabolome profiles are based on Bray‒Curtis dissimilarity with an ANOSIM to compare the metabolome profiles between (B) subjects and between (C) dietary terms.

To investigate the impact of daily dietary fluctuations on fecal metabolome profiles, ionic low-molecular-weight metabolites were measured by Capillary Electrophoresis Time-Of-Flight Mass Spectrometry (CE-TOFMS). The metabolome analysis detected 259 metabolites from 176 human fecal samples (Figure 3A). PCoA plots based on Bray‒Curtis dissimilarity in conjunction with ANOSIM of the fecal metabolome dataset showed that the profiles could be more clearly separated according to individuals rather than their dietary conditions (Figure 3B, C), as was the case for predicted metagenomic profiles (Figure 2E, F).

Using these metabolome data, we conducted a comprehensive correlation analysis between bacterial genera and metabolite concentrations. Correlations with an FDR > 0.1 based on Spearman's rank correlation coefficient were selected and visualized as a heatmap (Figure S4). As a result, positive or negative correlations were observed between multiple gut bacterial genera and metabolites such as *N*-acetylputrescine, taurocholate, sebacate, and azelate, which have previously been suggested to be accurately predictable from gut microbiota data.^19^

### Effects of dietary fluctuation on gut microbiome and metabolome profiles

**Figure 4.**
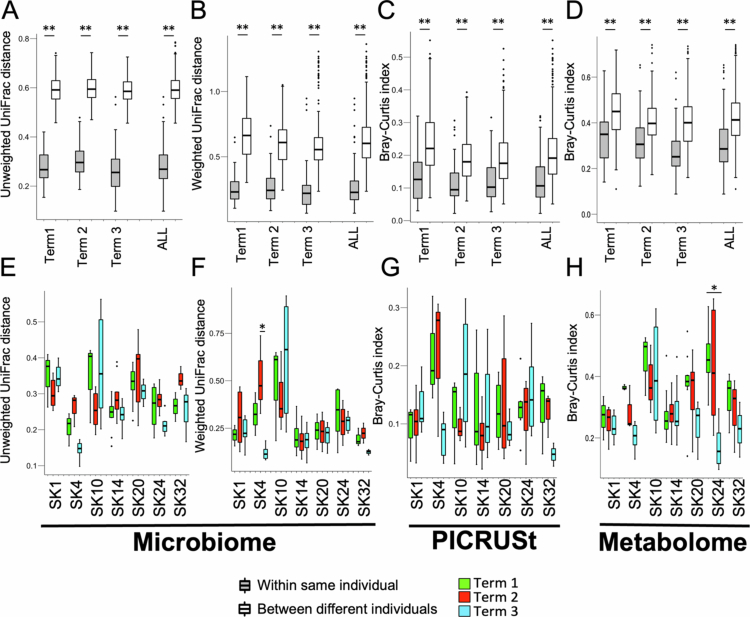
The human gut environment is robust under conditions of dietary fluctuation. (A–D) Comparison of microbiome profiles based on (A) unweighted or (B) weighted UniFrac distances, (C) predicted metagenome profiles, and (D) metabolome profiles based on Bray–Curtis dissimilarity. Comparisons were made within and between individuals for each term. (E–H) Comparison of microbiome profiles based on (E) unweighted and (F) weighted UniFrac distances and comparison of (G) PICRUSt-predicted functional metagenome profiles and (H) metabolome profiles based on Bray-Curtis dissimilarity within each individual for each term. Significant differences based on the Mann‒Whitney *U*-test (A‒D) or Steel‒Dwass test (E‒H) are indicated by *p < 0.05, **p < 0.01.

To statistically evaluate the effects of host dietary fluctuations in the gut microbiome and metabolome profiles, we selected seven subjects who provided at least three fecal samples during each phase for statistical analysis (the selected subjects are indicated by asterisks in Table S1). The characteristics of these seven subjects are shown in Figure S5. The unweighted and weighted UniFrac distances of the microbiome profiles and Bray‒Curtis dissimilarities of the metagenome profiles within the same individual were significantly shorter than the distances between different individuals during each dietary term and when all terms were combined (Figure 4A–C). Additionally, the Bray‒Curtis dissimilarities of metabolome profiles within the same individual were also significantly lower than the distances between different individuals during each dietary term and when all terms were combined (Figure 4D). These results suggested that not only the human gut microbiome, but also the metabolome profile is unique to individuals, which is consistent with the ANOSIM results (Figures 2A–F, 3B, C).

Next, we compared the distances between samples from the same individuals during each phase. In the microbiome profiles, there were no significant intra-individual changes across the dietary phases in all subjects based on unweighted UniFrac distances, weighted UniFrac distances, and Bray‒Curtis dissimilarities for the metagenome profiles, except for one person: SK04, in weighted UniFrac distance (Figure 4E–G). Moreover, the *α*-diversity index (Chao1, Shannon, and Faith PD) did not significantly differ among the three terms according to the Tukey‒Kramer test. These results suggested that the structure of the human microbiome is generally robust enough to withstand expected dietary fluctuations in daily life. Additionally, in the metabolome profiles, there were no significant intra-individual changes across the dietary phases in any of the subjects except for subject SK24, based on Bray–Curtis dissimilarity (Figure 4H). This suggested that, like the microbiome profile, the human fecal metabolome profile is also similarly robust.

In the analyses based on the weighted UniFrac distances of the gut microbiome and the Bray‒Curtis dissimilarity of the metabolome, one subject in each case showed a significantly shorter distance in samples from the third phase relative to those from the first and second phases. These differences occurred in different individuals for each respective measurement (SK04 and SK24, Figure 4F, H), suggesting that the stability of the gut microbiome is not necessarily correlated with the stability of the metabolome. Using the data from Term 1, which was designed to measure general gut environmental stability under conditions most similar to each subject's daily routine, we examined the association between metabolome stability, microbiome stability, dietary stability, and microbiome diversity. There were no significant correlations between microbiome stability and metabolome stability (Table S3 and Figure S6). The most significantly correlated factors were metabolome stability and Faith PD, a measure of *α*-diversity of the microbiome (correlation coefficient = –0.693, *q* = 0.038; see Table S3 and Figure S7).

As one individual (SK24) exhibited a more stable metabolite profile during Term 3, in which the same metabolites were consumed in each meal, we hypothesized that some metabolites in the feces that are more affected by host dietary fluctuations exist. To investigate these potentially affected metabolites, we screened for fecal metabolites showing significantly smaller variance during Term 3 compared to during Terms 1 and 2 by Bartlett’s test and F-test to compare variances between each term (Table 1). These metabolites accounted for 4%–25% of all detected metabolites. Additionally, the number of metabolites expected to be affected by host dietary fluctuations was highest in SK24 among the seven subjects, which is consistent with the distance analysis (Figure 4H). Moreover, no metabolites showed significantly small variance during Term 3 among all seven subjects. Thus, alterations in the fecal metabolite content in response to host dietary fluctuations varied among individuals.

**Table 1.** Metabolites that differed significantly during term 3 as compared with Terms 1 and 2.

	Subject ID (number of significantly different metabolites)						
	SK01	SK04	SK10	SK14	SK20	SK24	SK32
Metabolite	(12)	(29)	(10)	(21)	(31)	(63)	(31)
Adipate			*		*	*	*
Phenaceturate		*	*			*	*
2-Aminobenzamide		*			*	*	*
7,8-Dihydrobiopterin			*		*	*	*
5-Hydroxylysine	*	*				*	
Glucosaminate	*				*	*	
Tyrosine		*	*		*		
Leucine		*		*		*	
Betaine		*			*	*	
Cysteate		*			*	*	
2-Hydroxyoctanoate		*				*	*
Phenethylamine			*	*		*	
*N*-Formylaspartate			*			*	*
UDP-*N*-acetylglucosamine				*	*		*
Creatinine					*	*	*
Proline betaine	*			*			
Purine riboside	*			*			
beta-Alanine		*		*			
6-Hydroxynicotinate		*		*			
Tryptamine		*			*		
Pyridoxine		*				*	
Glycine-Glycine		*				*	
Tryptophan		*				*	
Guanine		*					*
1-Aminocyclopropane−1-carboxylate		*					*
gamma-Guanidinobutyrate			*			*	
*N*-Acetylleucine			*				*
Phenylalanine			*				*
Pyridoxamine 5'-phosphate				*	*		
Isoleucine				*		*	
Spermine				*		*	
Spermidine				*		*	
Threonine					*	*	
Creatine					*	*	
Glycine					*	*	
Methanesulfonate					*	*	
Taurocholate					*	*	
Cholate					*	*	
Phosphorylcholine					*	*	
Succinate					*	*	
*o*-Acetylcarnitine					*	*	
Carnitine					*	*	
Isopropanolamine					*	*	
1-Methylhistamine					*		*
*N*-Acetylglucosamine 6-phosphate					*		*
Octanoate					*		*
Itaconate					*		*
Valine						*	*
Carnosine						*	*
2-Hydroxypentanoate						*	*
Decanoate						*	*
5-Methylcytosine						*	*
Isethionate						*	*
Histidine						*	*
Acetylcholine						*	*
*N*,*N*-Dimethylglycine	*						
Serotonin	*						
2-Aminophenol	*						
*O*-Acetylserine	*						
Nicotinate	*						
Azelate	*						
Adenosine monophosphate	*						
*N*-Acetylglucosamine 1-phosphate	*						
*N*-Acetylornithine		*					
*N*-Acetyl-beta-alanine		*					
Heptanoate		*					
Pipecolate		*					
3-Aminoisobutyrate		*					
P1,P4-Di(adenosine−5') tetraphosphate		*					
Thiamine		*					
Pantothenate		*					
7-Methylguanine		*					
3-Methylhistidine		*					
*p*-Hydroxyphenylacetate		*					
gamma-Butyrobetaine		*					
*N*-Methylglutamate		*					
Histamine			*				
2,4-Diaminobutyrate				*			
Betaine aldehyde				*			
Choline				*			
Putrescine(1,4-Butanediamine)				*			
*N*-Acetylputrescine				*			
*N*-epsilon-Acetyllysine				*			
Uracil				*			
Alanine-Alanine				*			
Lysine				*			
Synephrine				*			
*cis*-Aconitate					*		
Sorbitol 6-phosphate					*		
Histidinol					*		
3-Methylguanine					*		
Glycocholate					*		
Allantoate						*	
5-Aminovalerate						*	
Guanidinoacetate						*	
Guanidinosuccinate						*	
Inosine						*	
Malonate						*	
Agmatine						*	
Uridine						*	
Cytidine						*	
alpha-Aminoadipate						*	
Nornicotine						*	
Digalacturonate						*	
beta-Imidazolelactate						*	
5-Methylthioadenosine						*	
5-Oxoproline						*	
1-Methyladenosine						*	
Trehalose 6-phosphate						*	
Alanine						*	
Ophthalmate						*	
Dihydrouracil						*	
2-Hydroxy−4-methylpentanoate						*	
Taurine						*	
Lactate						*	
1-Methylnicotinamide						*	
Indole−3-acetamide							*
Dopamine							*
*N*-Acetylphenylalanine							*
*N*-Methylalanine							*
Serine							*
DOPA							*
4-(beta-Acetylaminoethyl)imidazole							*

Taken together, these results indicated that the human fecal metabolome and microbiome profiles are unique for individuals and are robust with respect to daily dietary fluctuations in many cases. However, some fecal metabolites could be affected by these dietary fluctuations.

### Random forest classification can identify individuals from human fecal metabolome and metagenome profiles

**Figure 5.**
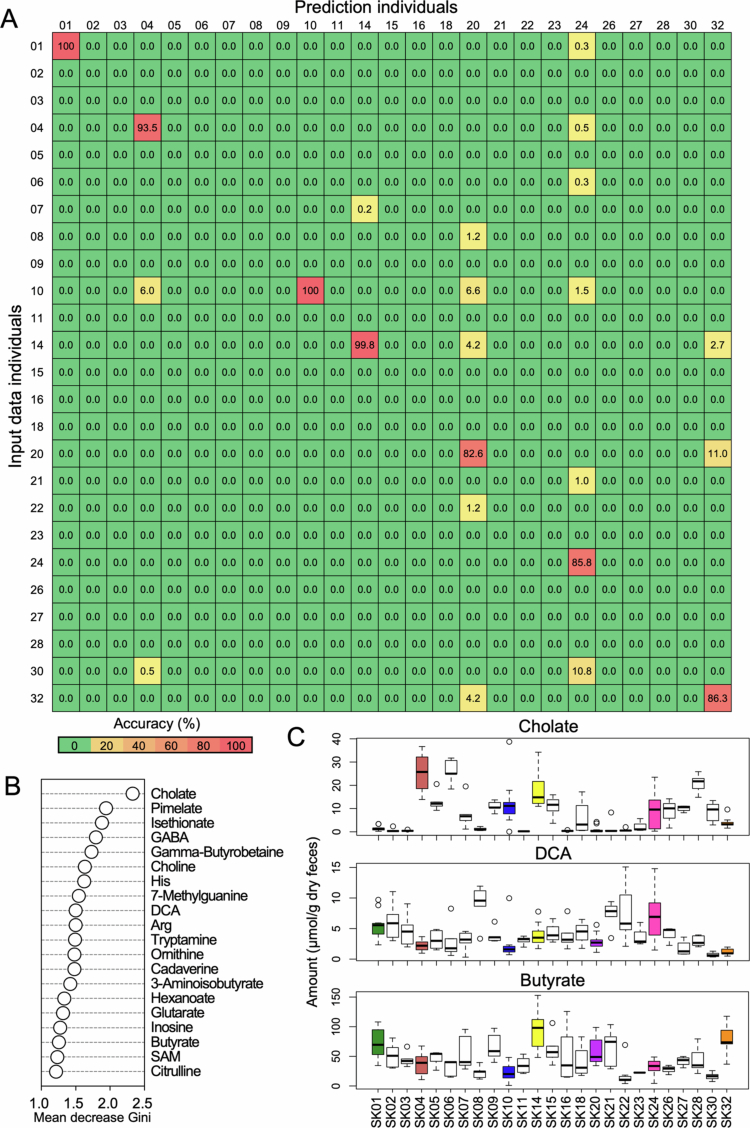
Random forest classification predicts the identity of individuals based on their fecal metabolome profiles. (A) Confusion matrix for evaluating prediction accuracy using fecal metabolome profiles. The random forest model was trained on data from all 25 individuals, and classification accuracy was evaluated on a subset of samples from seven individuals (SK01, 04, 10, 14, 20, 24 and 32) who had a sufficient number of samples. (B) The mean decrease Gini of subject identification using metabolome profiles. (C) Boxplots of metabolites that contributed to the identification of individuals.

As the distance analysis showed that the human gut microbiome and metabolome profiles were unique in individuals and generally stable, we next determined whether we could predict the identities of individuals by using their metabolome profiles. We used the random forest algorithm with the human fecal metabolome profiles. The random forest is a classification method well-suited for life sciences with strengths in handling high-dimensional data, performing feature importance analysis, and reducing overfitting.^20^^,^^21^ Because only seven individuals (indicated by asterisks in Table S1) had a sufficient number of samples to allow for both training and testing, we used 66% of the samples from these individuals for training and 33% for testing. For the remaining 18 individuals who did not have enough samples for separate evaluation, all of their samples were included in the training set. As a result, the model was trained on data from all 25 individuals, and the classification accuracy was evaluated on a subset of samples from the seven individuals. This approach yielded an overall accuracy of 92.69% (Figure 5A). This result was similar to that of the predicted metagenome profiles (total accuracy = 92.55%; Figure S8A). The top 20 metabolites that contributed most to accuracy, as based on the mean decrease Gini of the random forest analysis, are shown in Figure 5B. Some metabolites produced with the involvement of the gut microbiota, such as cholate, deoxycholic acid (DCA), and butyrate (Figure 5C), were among the factors contributing to the random forest classification. In this analysis, based on PICRUSt-derived metagenomic data, fatty acid and lipid biosynthesis, fermentation, and amine and polyamine biosynthesis were included in the top 20 pathways, representing factors contributing the most to accuracy (Figure S8B, C). These results indicated that random forest classification could predict each individual from their fecal metabolome profiles as well as metagenome profiles.

### The responses of intestinal environmental factors to daily dietary fluctuation varied across individuals

**Figure 6.**
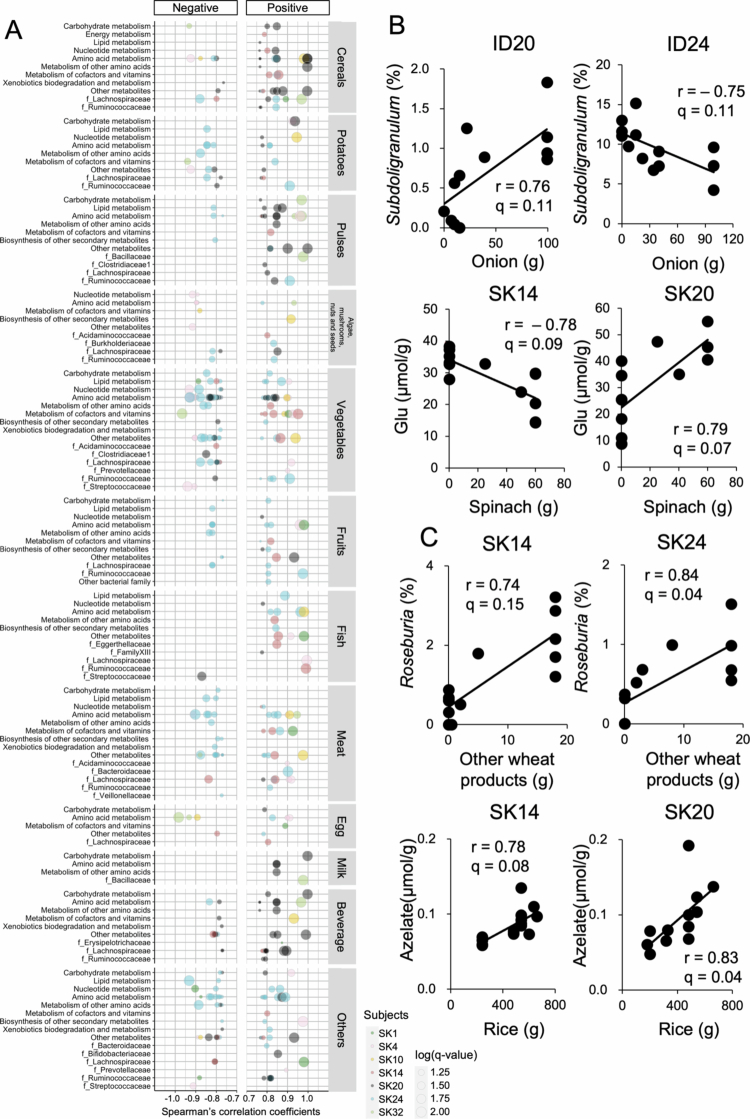
Changes in response to daily dietary fluctuations varied according to each individual. (A) Bubble-plot visualization of within-subject correlations between specific foods and genus-level taxonomy or metabolites (Spearman’s correlation; FDR-corrected *p* < 0.1). For visual simplicity, food choices, genus-level microbiome data, and metabolites are displayed according to their assignments in food categories, taxonomic families, and metabolic pathways, respectively. Both negative and positive correlations are shown. Stronger correlations are located on the outer edges of the plot. The size of each bubble corresponds to the log (FDR-corrected *p*-value), with larger bubbles representing smaller FDR-corrected *p*-value. Bubbles are colored by subject. (B and C) Two examples of Spearman’s correlations between food choices and genus-level microbiome data or food choices and metabolites with (B) opposite directionality across individuals and (C) two examples of conserved directionality are shown (all FDR-corrected *p* < 0.2). See Table S4 for the complete list of significant FDR-corrected *p*-values and correlations observed in more than two subjects.

As the human gut microbiome and metabolome profiles were highly personalized and the features of each individual were maintained even if the subjects ate the same diet, we hypothesized that individuals may have their own unique response to a given dietary component, which is consistent with a recent report.^12^ To test this hypothesis, we conducted correlation analyses between the intake of each food component and the relative abundances of genus-level microbial taxa and the amount of fecal metabolites by using the data from the seven selected participants. Visualization of the most significant correlations (false discovery rate [FDR] < 0.1) by food category, family-level taxonomy, and metabolic pathway revealed several interesting patterns (Figure 6A). As previously reported in shotgun metagenomic data,^12^ some subjects possess multiple genera and/or metabolites within the same family and/or pathway that are correlated with the intake of foods from the same food category. Among the seven subjects, SK24 showed the greatest number of correlations, suggesting that the individual’s gut microbiome and metabolome were most likely affected by dietary fluctuations (Figure 4H). Cereal consumption and fish consumption were significantly and positively correlated with several genera and metabolites in common across some subjects, suggesting some conservation of these relationships across individuals. Among the significant correlations between food and genus or between food and metabolites, 410 were found in more than one participant, whereas 50 were found in more than two participants (FDR < 0.1; Table S4). Consistent with the findings of a previous study,^12^ the directionality of not only these food‒genus relationships but also food‒metabolite relationships was not always conserved across individuals. For example, of the 50 significant correlations found in more than two participants, 12 correlations was in opposite directions (two examples are shown in Figure 6B), whereas the directionality of 38 correlations was conserved across individuals (two examples are shown in Figure 6C).

## Discussion

We investigated the uniqueness and stability of the human gut microbiome and metabolome in relation to dietary intake by analyzing data from 25 subjects under three different dietary conditions. Our findings revealed that both the microbiome and metabolome profiles were highly individual-specific and remained robust despite daily dietary fluctuations. Even when consuming identical diets, individuals retained their unique metabolome features, allowing identification with over 92% accuracy using a random forest algorithm.

Concerning the microbiome profiles, our results are consistent with previous studies, such as the finding that human gut microbiome structures are affected mainly by long-term dietary habits^22^ and that consuming a fermented milk product containing probiotic bacterial species has no significant effects on the human gut microbiome structure.^16^ With respect to metabolome profiles, only a few studies have investigated the inter- and intra-individual differences in fecal metabolome profiles in healthy subjects. Smirnov et al., ^23^ reported that primary results obtained by hierarchical clustering analysis of the data corresponding to operational taxonomic unit counts and the fecal metabolome showed that the profiles deriving from the same individuals tended to cluster together.^23^ Sillner et al., ^24^ also suggested that even in infants consuming the same type of milk (breast milk, formula, or formula with probiotics), individual differences in metabolome profiles in the stool are maintained.^24^ Additionally, a recent study conducted in Norway reported that host identity, rather than diet, is the dominant contributor to variability in fecal metabolome profiles.^13^ Furthermore, compared to studies conducted in the United States,^12^ Japanese subjects tend to have a greater proportion of carbohydrate intake (Figure 1C). The three most commonly consumed food items among Japanese participants were rice, dashi (a traditional Japanese stock made from dried bonito flakes and kelp, categorized under “Other seasoning”), and tea (such as green tea) (Figure 1B), reflecting dietary habits distinct from those of Americans. Despite these differences in dietary composition, our findings, in agreement with those of previous studies, suggest that individual differences in the metabolome are more pronounced than the influence of diet. This finding indicates that the relative stability of fecal metabolome profiles is a commonly shared trait across populations, regardless of dietary habits and ethnicity. Additionally, our study demonstrated that healthy Japanese adults maintained personalized fecal metabolome profiles even when consuming the same diet, further supporting the robustness of this individual-specific metabolic signature.

To compare the robustness of the human microbiome and metabolome structure, we compared the diversity of the microbiome, predicted metagenome, and metabolome profiles between samples from each individual during the three dietary phases. There were no significant intra-individual changes in any of the unweighted or weighted UniFrac distances or Bray‒Curtis dissimilarity of predicted metagenome values across dietary phases, with the exception of one person with respect to the weighted UniFrac distance. The analysis of metabolome profiles revealed no significant changes between samples from the same individual during each dietary phase in six of seven subjects on the basis of Bray‒Curtis dissimilarity. This result supports the hypothesis that human fecal metabolome profiles are robust against daily dietary fluctuations, suggesting that the stability of the human microbiome structure and metabolome is often unaffected by changes in the diet that normally occur in daily life. Switching from an animal-based diet to a plant-based diet and vice versa quickly alters the human gut microbiome composition and concentration of some SCFAs.^10^ The robustness of the microbiome and metabolome profiles obtained in our study may reflect the relative insignificance of fluctuations that occur in our diets within our daily lives.

In the analyses based on the weighted UniFrac distance of the microbiome and the Bray‒Curtis dissimilarity of the metabolome, a single subject in each metric had significantly shorter distances between samples associated with Term 3 than those associated with Terms 1 or 2. When we examined the relationships between metabolite stability, microbiome stability, diet stability, and microbiome diversity, we found no significant correlation between microbiome stability and metabolome stability. The most significantly correlated factors were metabolite stability and Faith PD (correlation coefficient = –0.693, *p* = 0.038). Low diversity in the gut microbiome is one of the defining traits of dysbiosis, and studies have shown that people with inflammatory bowel disease,^25^ obesity,^18^ type 1 and 2 diabetes,^26^^,^^27^ atopic eczema,^28^ celiac disease,^29^ and arterial stiffness^30^ have lower intestinal bacterial diversity than healthy controls. In addition, greater gut microbiome richness is associated with increased microbiome stability in response to increased dietary fiber intake.^31^ This suggests that the diversity of the gut microbiome may contribute to the homeostasis that allows stable metabolites to be produced, rather than the stability of the gut microbiome.

When comparing between individuals, SK24 and SK10 tended to have relatively unstable metabolome profiles (Figure 4H, Figure S6). These two individuals also exhibited relatively low *α-*diversity (Figure S6), and this result aligns with the correlation analysis shown in Figure S7. On the other hand, the difference between these two individuals lies in the fact that while the metabolite profile of SK24 stabilized during Term 3, when they continued the same diet, SK10 maintained a relatively unstable profile similar to that of Term 1 and Term 2. This finding suggests that even individuals with relatively unstable metabolite profiles may have factors that are either associated with dietary fluctuations or not. Interestingly, there was also individual variation in the stability of metabolome profiles even when the same diet was used. While it is difficult to definitively prove what causes this in this study, it is possible that microbiota diversity, which is the factor most strongly correlated with the stability of metabolome profiles, or the factors that were not monitored in this research, such as chewing frequency and lifestyle patterns. Further studies are needed to pinpoint the precise cause of these variations.

Although in many cases, the human fecal metabolome profiles were unique to individuals and the characteristics of the fecal metabolome profiles were maintained under dietary variation, our screening results suggested that some metabolites were influenced by these dietary variations. This screening revealed no common metabolites for which the variance was significantly small during Term 3 among the seven subjects examined. One of the most common metabolites, adipate, was significantly lower when the hosts ate the same meals than when the hosts ate different meals in four of the seven subjects. Adipate is added to foods as an acidulant and gelatinizer.^22^ Significant changes in the variance of this metabolite were observed in only four of seven subjects, but the results may have been affected by the frequency with which processed foods were consumed that contained food additives. This result suggests that our technique detected a food-derived metabolite in fecal metabolome profiling.

Some metabolites may have been affected by the dietary components ingested by the host. However, the metabolome profiles maintained their differences at the individual level. In fact, random forest classification was able to predict individual-level differences with > 92% accuracy according to the unique features of the metabolome profiles. Individual identities have previously been predicted by using the gut microbiome composition,^17^ metagenomic profiles,^17^ and skin microbiome profiles.^21^^,^^32^ To the best of our knowledge, our study is the first to report the successful identification of individuals by using a random forest approach with human fecal metabolome profiles. Moreover, this algorithm could predict the identity of individuals even though the model used a dataset corresponding to information from all three terms, supporting the idea that metabolome profiles are robust under conditions of daily dietary fluctuations. The dataset used in this study is relatively small, and further research is needed to determine whether the results are reproducible in other datasets. However, the finding that the characteristics of the fecal metabolome are unique to individuals is consistent with the findings of previous studies.^13^^,^^23^ The application of random forest analysis enables the identification of individual-specific characteristics of the gut environment, allowing for the determination of key parameters that reflect unique host features. These parameters may be utilized for predicting drug efficacy, assessing physiological traits, and recommending appropriate dietary interventions. Furthermore, if the relationship between the gut microbiota composition and disease susceptibility is elucidated, this approach may contribute to the prediction of disease risk and the development of personalized healthcare strategies.

We note that, in this study, ionic low-molecular-weight metabolites were analyzed to focus on low-molecular-weight metabolites produced by the gut microbiota and host as degradation products of the diet, whereas compounds not suitable for CE-TOFMS analysis, such as highly polymerized substances, such as polysaccharides, as well as lipids, polyphenols and isoflavones, were excluded. Further investigations are needed to determine whether the substances excluded in this study were affected by diet. Additionally, host physiology factors such as pH and the female menstrual cycle may affect metabolome profiles. Therefore, further research is needed to address these points. Notably, the participants in this study were all Japanese, which may limit generalizability. In addition, a limitation of this study is the relatively small sample size, as only a limited number of participants were able to provide three or more samples within the 5- to 7-d period. We are not able to draw robust conclusions owing to these limitations, but we believe that there is value in presenting the trends and observations of our study, which present novel observations of human microbiota and metabolome changes over time.

In recent years, studies of human fecal metabolome profiles of healthy and diseased patients have yielded useful insights into disease prevention and treatment.^33-36^ In particular, methods are being developed to distinguish the disease state from the healthy state using gut environmental datasets, including fecal metabolome data, in conjunction with machine learning.^37-40^ The findings from this study suggest that the influence of the diet consumed by the subject immediately prior to sample collection is not significant when obtaining such data, which should help reduce barriers for further research on the gut environment. Moreover, our results indicate that individuals exhibit distinct metabolic responses to the same diet, suggesting that targeted nutritional interventions may require personalized optimization. This highlights the potential application of our findings in precision nutrition, where dietary strategies can be tailored to maximize specific health benefits for each individual. Taken together, our findings provide insight into the understanding and utilization of fecal metabolome information for the advancement of human health.

## Materials and methods

### Sample collection

In this study, fecal samples were obtained from 25 healthy Japanese subjects under three different dietary conditions. This study was approved by the Ethical Committees of Keio University Shonan Fujisawa Campus (No. 195, R000050505 UMIN000044571). Subjects with intestinal diseases, skin diseases, those who had taken antibiotics, or allergies to the specified diet were excluded. All the subjects were informed of the purpose of this study, and written consent was obtained from all the subjects. This study was conducted with strict consideration for privacy. The subject information and number of fecal samples obtained from each subject are shown in Figure S1B. During the first phase (Term 1, 7 d), there was no restriction on diet, but the consumption of medicinal drugs and dietary supplements was prohibited. During the second phase (Term 2, 7 d), the subjects ate the provided meals, which differed for each mealtime. During the last phase (Term 3, 5 d), the subjects repeatedly ate the identical meal 15 times, with three meals a day over a 5-d period. For the remaining 2 d of this term, the subjects were under the same conditions as those in Term 1. During Term 1, the subjects recorded their meals by using a recording form and/or taking pictures. Commercially available boxed meals, frozen foods, fruits, sandwiches, rice balls, and retorted foods were provided in Terms 2 and 3. The menus of the meals provided to the subjects during Term 3 are indicated in Figure S1B. There were two types of menus, one of which was assigned to each subject depending on the residential district of the subject (see Figure S1B). Throughout the trial, the subjects collected their feces and froze them as soon as possible. The frozen fecal samples were shipped to the Institute for Advanced Biosciences, Keio University, Yamagata, Japan, and stored at -80°C. Fecal samples were lyophilized for at least 10 h using a VD-800R lyophilizer (TAITEC, Japan) for metabolome and microbiome analysis.

### Fecal metabolite extraction and CE-TOFMS measurements

Metabolites in the fecal samples were analyzed as previously described.^41^^,^^42^ Briefly, fecal metabolites were extracted from ~10 mg of freeze-dried feces by vigorous shaking with 500 μL of methanol containing 20 μM each of methionine sulfone (Alfa Aesar, A17027) and d-camphol−10-sulfonic acid (CSA) (Fujifilm Wako, 4987481429680) as the internal standards. The mixture was combined with ~100 mg of 0.1-mm zirconia/silica beads (BioSpec Products, USA) and subjected to 5 min of vigorous shaking using a Shake Master Neo (Biomedical Science, Japan). Then, the mixture was subsequently extracted with 500 μL of chloroform and 200 μL of water. The suspension was centrifuged at 4,600 × *g* for 15 min at 4 °C, and the resulting supernatant was transferred to a 5-kDa-cutoff filter column (Ultrafree MC-PLHCC 250/pk for metabolome analysis; Human Metabolome Technologies, Japan). The flow-through was dried under vacuum, and the residue was then dissolved in 50 μL of Milli-Q water containing reference compounds (200 μM each of 3-aminopyrrolidine and trimesate). The levels of the extracted metabolites were measured in both positive and negative modes by CE-TOFMS as previously described.^43^ All the CE-TOFMS experiments were performed using an Agilent capillary electrophoresis system (Agilent Technologies, USA).

### Metabolome data processing and analysis

The raw data were analyzed using our proprietary automatic integration software MasterHands (ver. 2.16.0.15).^43^ Annotation tables were produced from measurements of standard compounds and were aligned with the datasets according to similar *m*/*z* values and normalized migration times. The peak areas were subsequently normalized against those of the internal standards methionine sulfone and CSA for cationic and anionic metabolites, respectively. The amounts of each metabolite were calculated based on their relative peak areas and the concentrations of the standard compounds. All samples were rearranged in random order for extraction and measurement to reduce the technical variations between batches of measurements. The quality control samples were also measured to check the differences between batches, with reference to a previous study.^44^ HCA was performed by Pearson correlation. These analyses were performed using MeV TM4 software (ver. 4.8.1; Dana-Farber Cancer Institute**,** USA).^45^

### DNA extraction

Fecal DNA isolation was performed as previously described^46^ with some modifications. In brief, each freeze-dried fecal sample was combined with four 3.0-mm zirconia beads, ~100 mg of 0.1-mm zirconia/silica beads, 400 μL of DNA extraction buffer (TE containing 1% [w/v] sodium dodecyl sulfate), and 400 μL of phenol/chloroform/isoamyl alcohol (25:24:1, [v/v/v]) and was then subjected to vigorous shaking (1500 rpm for 15 min) using a Shake Master Neo. The resulting emulsion was subjected to centrifugation at 17,800 × *g* for 10 min at room temperature, and bacterial genomic DNA was purified from the aqueous phase using a standard phenol/chloroform/isoamyl alcohol protocol. RNA was removed from the sample by RNase A treatment; the resulting DNA sample was then purified again by another round of phenol/chloroform/isoamyl alcohol treatment.

### 16S rRNA gene sequencing

16S rRNA genes in the fecal DNA samples were analyzed using the MiSeq sequencer (Illumina, USA). The V1–V2 region of the 16S rRNA genes was amplified from the DNA isolated from feces the bacterial universal primer set 27Fmod (5'-AGRGTTTGATYMTGGCTCAG-3') and 338 R (5'-TGCTGCCTCCCGTAGGAGT-3’).^47^ PCR was performed with Tks Gflex DNA Polymerase (Takara Bio Inc., Japan), and amplification proceeded with one denaturation step at 98 °C for 1 min, followed by 20 cycles of 98 °C for 10 s, 55 °C for 15 s, and 68 °C for 30 s, with a final extension step at 68 °C for 3 min. The amplified products were purified using Agencourt AMPure XP (Beckman Coulter, USA) and then further amplified using the forward primer 5'-AATGATACGGCGACCACCGAGATCTACACNNNNNNNNTATGGTAATTGTAGRGTTTGATYMTGGCTCAG-3', which contains the P5 sequence, a unique 8-bp barcode sequence for each sample (indicated by ‘N’), the Rd1SP sequence, and the 27Fmod primer, and the reverse primer 5'-CAAGCAGAAGACGGCATACGAGATNNNNNNNNAGTCAGTCAGCCTGCTGCCTCCCGAGGAGT-3', which contains the P7 sequence, a unique 8-bp barcode sequence for each sample, the Rd2 SP sequence, and the 338 R primer. After purification using Agencourt AMPure XP kits, the purified products were mixed at approximately equal molar concentrations to generate a 4 nM library pool, from which the final library pool was diluted to 6 pM, including a 10% PhiX Control v3 (Illumina, USA) spike-in for sequencing. Finally, MiSeq sequencing was performed. In this study, 2 × 300-bp paired-end sequencing was used.

### Analysis of 16S rRNA gene sequences using QIIME 2

Analysis of 16S rRNA gene sequences was performed as described previously^48^ with some modifications. In brief, filter-passed reads were processed using Quantitative Insights into Microbial Ecology (QIIME) 2 (2019.10.0).^49^ Denoising and trimming of sequences were carried out using DADA2. The first 20 bp and 19 bp were trimmed from the 5’ end of both forward and reverse reads, respectively, to remove primer sequences. The resulting 135-bp and 220-bp reads from the respective 5’ ends were used for subsequent steps. Taxonomy was assigned using the SILVA132 database^50^^,^^51^ using the Naive Bayesian Classifier algorithm. The alpha diversity of the gut microbiota was analyzed using observed species, Faith PD, and Shannon indices. PCoA based on UniFrac distances and ANOSIM was carried out using QIIME 2. Metagenome predictions were generated with the PICRUSt2^52^ plugin under QIIME 2.

### Dietary information

We distributed food logs to each subject for self-reporting of every food item that was consumed during the study period. Logs were returned at the end of the study. Each subject was asked to record the time and amount of food consumed and/or take photos of their food against a grid background of defined size to indicate the amount of food they consumed. The intake of each nutrient and ingredient was calculated using commercially available software (Excel Eiyoukun version 4.0; Kenpakusya, Japan). For food category analysis, the nutritional content of 2,294 foods listed in the ''Standard Tables of Food Composition in Japan 2015, 7th revised and enlarged edition (Japanese Food Composition Table) was used. For food choice analysis, the 98 food groups used to categorize the 2,116 food items used in the The Japan National Health and Nutrition Survey were used for the correlation analysis of food intake patterns.

### Statistical analysis

The Spearman’s rank correlation coefficients of every pair of abundance of genus and metabolome concentrations were calculated using R; pairs that exhibited significant correlation (FDR < 0.1 based on Benjamini‒Hochberg correction) were displayed in a heatmap with HCA based on Pearson correlation by using MeV TM4 software (ver. 4.8.1; Dana-Farber Cancer Institute**,** USA).^45^ The non-parametric Mann–Whitney *U*-test and Steel‒Dwass test with FDR values based on the Benjamini‒Hochberg correction were used for statistical evaluations of distance comparisons between two groups and multiple groups. To find those metabolites for which their variances were significantly smaller in Term 3 than in Terms 1 and 2, we used Bartlett’s test and the F-test. ANOSIM was used to evaluate the similarities between samples. ANOSIM was carried out with the QIIME 2 pipeline, and the number of permutations was 999. For correlation analysis, Spearman’s rank correlation was used.

### Random forest analysis

For the random forest classifier, the fecal metabolome or predicted metagenome data from all 25 subjects for all three terms were used. For the selected seven subjects (see Table S1), 66% of their samples were used for model training and 33% were used for testing. The training and test samples were randomly split. For the remaining 18 subjects, all samples were used solely for model training and were not included in the test set. The accuracy rates and mean decrease Gini score were calculated 100 times, and the score average was used for evaluation. A random forest method was then implemented with the randomForest function in the randomForest package v4.6-14 in R. In each test, 5,000 decision trees were generated.

### Data accession

The microbiome analysis data have been deposited in the DDBJ Sequence Read Archive (http://trace.ddbj.nig.ac.jp/dra/) under accession number DRA014479. The metabolome data are available at the NIH Common Fund's National Metabolomics Data Repository (NMDR) website, the Metabolomics Workbench, https://www.metabolomicsworkbench.org, where they have been assigned Project ID (PR001413**)**. The data can be accessed directly via the Project DOI: http://dx.doi.org/10.21228/M82X3Z. This work is supported by the National Institutes of Health grant U2C-DK119886.

## Supplementary Material

Supplementary material Supplementary_Figures_high_reso.

Supplementary material Supplementary_Figure_legends_25110505_Nov_2025_23_40.

Supplementary material Tables_25110505_Nov_2025_23_41.
