# Supplementary material for: Ecologically robust gut environment associated with personalized metabolic responses in a Japanese cohort
Source: Gut Microbes Rep. 2025 Nov 16;2(1):2574930. doi: 10.1080/29933935.2025.2574930 (PMC12940129; doi:10.1080/29933935.2025.2574930)
Supplement: Supplementary material — Supplementary_Figure_legends_25110505_Nov_2025_23_40. [file KGMR_A_2574930_SM4620.docx]

**Fig. S1. Study design overview and sampling schedule.**

(A) Twenty-five Japanese individuals were recruited for this study. They each underwent three diet phases as follows. During the first 7 days (Term 1, indicated in green), there were no dietary restrictions, but consumption of medicinal drugs and dietary supplements was prohibited. During the next 7 days (Term 2, indicated in red), the individuals consumed meals provided to them. The menu for each meal was different. For the last 7 days (Term 3, indicated in blue), individuals were provided an identical meal 15 times (i.e., three times per day for 5 days). (B) Detailed sampling schedule for each subject. The circles indicate time points when individuals provided fecal samples. The meal type for Term 3 is shown under the heading “Meal”: T, tofu patty meals that consisted of a tofu patty with sauce, *kinpira gobo* (burdock root), *kabocha* (Japanese pumpkin) squash with soy sauce, pork and spinach with sesame sauce, miso soup, steamed white rice, and a banana; M, meat patty meals that consisted of a meat patty with demi-glace sauce, macaroni gratin, caponata, miso soup, steamed white rice, and a banana. The images of the two meal types were indicated at the bottom of this Figure.

**Fig. S2. Relative abundance in microbiome profiles.**

Bar graph showing the relative abundance of the top 16 most abundant genera in all subjects. Labels at the bottom of the graph indicate the terms and subjects. The microbiome profiles differed across individuals but were relatively consistent over time within each individual.

**Fig. S3. Functional metagenome profiles predicted by PICRUSt.**

Bar graph showing the relative abundance of KEGG pathways as predicted using PICRUSt in all subjects. Labels at the bottom of the graph indicate the terms and subjects. The metagenome profiles were more similar across individuals, rather than microbiome profiles.

**Fig. S4. Heatmap of relationships between metabolites and bacterial genera.**

A heatmap illustrating the correlations between metabolites and bacterial genera based on Spearman’s rank correlation coefficients. Correlations were calculated using all samples, and only those with an absolute correlation coefficient of 0.4 or higher and an FDR-corrected p-value lower than 0.1 were selected. Gray indicates cases where either the correlation coefficient or the FDR did not meet the selection criteria. Red and blue represent positive and negative correlations, respectively. Hierarchical clustering was performed based on Pearson correlation.

**Fig. S5. Analysis of data from the seven subjects who provided three or more fecal samples per term as related to Fig. 1–3.**

**(A–G)** Data from the seven subjects are highlighted in PCoA plots based on Bray-Curtis dissimilarity of **(A)** food categories, food choices **(B)**, and nutrients **(C)**; unweighted UniFrac distances **(D)** and weighted UniFrac distances **(E)** of microbiome profiles; and Bray-Curtis dissimilarity of PICRUSt-predicted metagenome profiles **(F)** and fecal metabolome profiles **(G)**. The selected seven subjects did not have outlier profiles compared to the other participants.

**Fig. S6. Stability of metabolome, microbiome, and food choices, and α-diversity of the microbiome during term 1.**

Box plots show the Bray-Curtis distance of metabolome and food choice profiles and UniFrac distance (weighted and unweighted) of microbiome profiles within each individual during term 1. The subjects who provided only a single sample during term 1 are shown as ‘no data’. The α-diversity of the microbiome during term 1 was evaluated using the observed species data and the Shannon and Faith PD indexes.

**Fig. S7. Correlation between stability of metabolome and Faith PD as related to Table S3.**

Scatter plot shows the correlation between the stability of the metabolome (Bray-Curtis distance of the metabolome during term 1 for each subject) and Faith PD during term 1 for each subject. Fifteen subjects who provided at least 2 samples in term 1 were used for this analysis.

**Fig. S8. Random forest classification predicts the identity of individuals based on predicted gut metagenome profiles**

(A) Confusion matrix for evaluating prediction accuracy using predicted metagenome profiles. The random forest model was trained using data from all 25 individuals, and classification accuracy was evaluated using a subset of samples from the seven individuals (SK01, 04, 10, 14, 20, 24 and 32) who had a sufficient number of samples.

(B) The mean decrease Gini of subject identification using predicated metabolome profiles.

(C) Boxplots of metabolites that contributed to the identification of individuals.
