# Supplementary material for: Ecologically robust gut environment associated with personalized metabolic responses in a Japanese cohort
Source: Gut Microbes Rep. 2025 Nov 16;2(1):2574930. doi: 10.1080/29933935.2025.2574930 (PMC12940129; doi:10.1080/29933935.2025.2574930)
Supplement: Supplementary material — Tables_25110505_Nov_2025_23_41. [file KGMR_A_2574930_SM4621.docx]

**Table S1. Information about the participants of this study**

|  |  |  | Subject information | |  | Number of fecal samples collected | | | |
| --- | --- | --- | --- | --- | --- | --- | --- | --- | --- |
| Subject ID | |  | Sex | Age (years) |  | Term 1 | Term 2 | Term 3 | Total |
| SK01 | * |  | Male | 26 |  | 3 | 4 | 4 | 11 |
| SK02 |  |  | Male | 25 |  | 2 | 2 | 2 | 6 |
| SK03 |  |  | Male | 25 |  | 1 | 1 | 3 | 5 |
| SK04 | * |  | Male | 26 |  | 3 | 3 | 4 | 10 |
| SK05 |  |  | Female | 28 |  | 1 | 2 | 2 | 5 |
| SK06 |  |  | Female | 28 |  | 1 | 2 | 2 | 5 |
| SK07 |  |  | Female | 36 |  | 1 | 2 | 2 | 5 |
| SK08 |  |  | Female | 35 |  | 1 | 2 | 2 | 5 |
| SK09 |  |  | Female | 35 |  | 1 | 2 | 2 | 5 |
| SK10 | * |  | Male | 33 |  | 3 | 4 | 4 | 11 |
| SK11 |  |  | Male | 29 |  | 2 | 2 | 1 | 5 |
| SK14 | * |  | Female | 36 |  | 5 | 5 | 5 | 15 |
| SK15 |  |  | Female | 27 |  | 2 | 2 | 2 | 6 |
| SK16 |  |  | Female | 29 |  | 2 | 2 | 2 | 6 |
| SK18 |  |  | Female | 30 |  | 1 | 2 | 2 | 5 |
| SK20 | * |  | Male | 30 |  | 5 | 5 | 3 | 13 |
| SK21 |  |  | Female | 28 |  | 2 | 2 | 2 | 6 |
| SK22 |  |  | Female | 39 |  | 2 | 3 | 3 | 8 |
| SK23 |  |  | Female | 27 |  | 1 | 1 | 1 | 3 |
| SK24 | * |  | Male | 37 |  | 4 | 4 | 4 | 12 |
| SK26 |  |  | Male | 23 |  | 1 | 1 | 1 | 3 |
| SK27 |  |  | Female | 25 |  | 1 | 1 | 1 | 3 |
| SK28 |  |  | Female | 55 |  | 2 | 1 | 2 | 5 |
| SK30 |  |  | Female | 30 |  | 4 | 3 | 2 | 9 |
| SK32 | * |  | Female | 42 |  | 3 | 3 | 3 | 9 |

**Table S2. Information about energy and nutrient of meals that participants consumed**

| Subject / Term | Day | Energy (kcal) | Protein (g) | Total fat (g) | Carbohydrate (g) | Total dietary fiber (g) |
| --- | --- | --- | --- | --- | --- | --- |
| SK01 | 1 | 2694.1 | 73.3 | 93.0 | 311.7 | 16.1 |
|  | 2 | 3197.5 | 136.8 | 93.9 | 199.7 | 12.3 |
|  | 3 | 1088.7 | 53.4 | 15.5 | 162.5 | 5.0 |
|  | 4 | 1739.8 | 65.2 | 57.7 | 191.3 | 7.1 |
|  | 5 | 3127.2 | 132.7 | 106.1 | 187.3 | 7.1 |
|  | 6 | 2607.3 | 91.6 | 91.2 | 282.2 | 14.7 |
|  | 7 | 1940.1 | 90.2 | 35.3 | 229.2 | 11.0 |
| SK02 | 1 | 2405.8 | 108.7 | 80.1 | 251.0 | 12.1 |
|  | 2 | 2118.0 | 71.7 | 41.4 | 299.6 | 13.6 |
|  | 3 | 2899.9 | 115.7 | 109.2 | 254.7 | 16.6 |
|  | 4 | 2763.1 | 111.6 | 98.2 | 294.6 | 12.0 |
|  | 5 | 2368.7 | 137.0 | 27.5 | 334.5 | 7.2 |
|  | 6 | 2897.2 | 137.0 | 86.8 | 312.6 | 20.4 |
|  | 7 | 4052.3 | 148.8 | 158.3 | 408.6 | 19.2 |
| SK03 | 1 | 2223.4 | 80.1 | 95.0 | 210.8 | 14.1 |
|  | 2 | 1752.6 | 76.8 | 64.9 | 172.4 | 10.8 |
|  | 3 | 1975.4 | 67.0 | 68.2 | 220.2 | 10.0 |
|  | 4 | 1397.2 | 62.8 | 53.9 | 133.5 | 7.9 |
|  | 5 | 1611.7 | 60.1 | 62.0 | 162.6 | 9.2 |
|  | 6 | 1399.5 | 61.5 | 48.5 | 155.4 | 9.5 |
|  | 7 | 2056.4 | 79.3 | 77.5 | 211.2 | 13.4 |
| SK04 | 1 | 1569.8 | 55.2 | 23.1 | 176.6 | 20.0 |
|  | 2 | 1884.5 | 65.4 | 52.0 | 207.3 | 9.4 |
|  | 3 | 1871.5 | 74.6 | 63.2 | 209.5 | 11.4 |
|  | 4 | 2754.5 | 103.3 | 110.1 | 288.8 | 11.9 |
|  | 5 | 2225.0 | 67.8 | 112.5 | 201.1 | 6.8 |
|  | 6 | 2542.6 | 70.2 | 105.4 | 250.2 | 10.6 |
|  | 7 | 2238.4 | 82.1 | 89.1 | 223.6 | 6.2 |
| SK05 | 1 | 1961.4 | 92.9 | 54.5 | 217.3 | 12.1 |
|  | 2 | 1768.5 | 49.8 | 57.3 | 215.8 | 11.9 |
|  | 3 | 1028.0 | 35.4 | 18.3 | 152.4 | 4.5 |
|  | 4 | 664.1 | 20.5 | 12.3 | 99.1 | 4.8 |
|  | 5 | 890.7 | 25.6 | 16.2 | 137.1 | 3.7 |
|  | 6 | 1833.0 | 67.7 | 26.8 | 258.3 | 11.4 |
|  | 7 | 1207.5 | 49.9 | 28.6 | 162.1 | 3.7 |
| SK06 | 1 | 1410.4 | 55.8 | 36.2 | 185.2 | 9.3 |
|  | 2 | 2526.4 | 91.2 | 91.5 | 256.7 | 8.0 |
|  | 3 | 3039.6 | 90.6 | 84.5 | 331.8 | 17.2 |
|  | 4 | 1352.2 | 47.0 | 21.3 | 201.2 | 8.3 |
|  | 5 | 1969.8 | 85.3 | 50.0 | 232.2 | 11.8 |
|  | 6 | 2745.9 | 108.1 | 94.4 | 279.3 | 11.4 |
|  | 7 | 2032.2 | 60.6 | 63.3 | 245.4 | 11.2 |
| SK07 | 1 | 1985.8 | 69.4 | 36.0 | 283.5 | 12.8 |
|  | 2 | 1646.3 | 56.8 | 38.2 | 212.7 | 4.9 |
|  | 3 | 1785.8 | 53.4 | 51.9 | 145.0 | 15.8 |
|  | 4 | 1555.8 | 55.3 | 36.8 | 214.0 | 6.6 |
|  | 5 | 1664.2 | 60.3 | 41.8 | 204.9 | 6.7 |
|  | 6 | 1860.2 | 42.0 | 76.1 | 122.5 | 6.6 |
|  | 7 | 2913.8 | 79.0 | 104.3 | 222.9 | 16.2 |
| SK08 | 1 | 1074.3 | 57.9 | 42.5 | 89.3 | 6.1 |
|  | 2 | 1491.2 | 39.1 | 55.0 | 121.5 | 5.6 |
|  | 3 | 645.1 | 27.8 | 13.1 | 4.4 | 2.9 |
|  | 4 | 1068.6 | 59.8 | 38.9 | 91.8 | 7.3 |
|  | 5 | 1078.6 | 29.4 | 28.3 | 138.4 | 8.8 |
|  | 6 | 1277.8 | 36.7 | 16.6 | 138.9 | 10.6 |
|  | 7 | 1595.7 | 47.0 | 42.5 | 154.2 | 7.3 |
| SK09 | 1 | 1478.3 | 56.4 | 56.6 | 111.3 | 10.8 |
|  | 2 | 1695.2 | 71.4 | 73.8 | 119.4 | 9.5 |
|  | 3 | 1826.5 | 77.9 | 58.8 | 137.0 | 14.1 |
|  | 4 | 1452.1 | 70.0 | 46.6 | 125.4 | 7.1 |
|  | 5 | 1036.5 | 31.0 | 26.4 | 117.4 | 6.7 |
|  | 6 | 1226.6 | 52.2 | 43.1 | 120.1 | 8.9 |
|  | 7 | 1673.4 | 52.0 | 78.1 | 106.7 | 9.9 |
| SK10 | 1 | 2444.6 | 102.7 | 67.9 | 243.6 | 13.5 |
|  | 2 | 2714.3 | 111.9 | 95.6 | 240.0 | 9.1 |
|  | 3 | 3014.9 | 118.4 | 86.9 | 343.4 | 22.9 |
|  | 4 | 2407.8 | 83.7 | 70.6 | 300.3 | 8.7 |
|  | 5 | 4357.5 | 131.9 | 138.1 | 496.6 | 28.4 |
|  | 6 | 3728.7 | 82.4 | 94.8 | 184.8 | 10.1 |
|  | 7 | 3190.7 | 158.2 | 62.9 | 372.1 | 18.8 |
| SK11 | 1 | 1783.4 | 93.2 | 110.3 | 66.2 | 9.6 |
|  | 2 | 1220.2 | 62.4 | 58.2 | 89.6 | 13.9 |
|  | 3 | 1216.2 | 68.8 | 47.9 | 94.0 | 13.5 |
|  | 4 | 859.9 | 48.2 | 21.6 | 91.3 | 14.7 |
|  | 5 | 1093.9 | 87.7 | 53.3 | 40.4 | 9.9 |
|  | 6 | 1836.4 | 23.3 | 33.7 | 95.2 | 13.4 |
|  | 7 | 756.8 | 57.4 | 43.5 | 13.5 | 9.6 |
| SK14 | 1 | 1731.4 | 58.1 | 54.7 | 209.5 | 10.1 |
|  | 2 | 1609.8 | 56.2 | 49.4 | 195.1 | 9.7 |
|  | 3 | 2443.5 | 94.9 | 104.2 | 217.6 | 19.2 |
|  | 4 | 1622.0 | 51.1 | 46.4 | 213.5 | 12.3 |
|  | 5 | 1993.7 | 62.4 | 39.1 | 294.7 | 13.3 |
|  | 6 | 1439.9 | 56.5 | 41.1 | 148.3 | 10.0 |
|  | 7 | 3304.7 | 92.5 | 94.4 | 422.9 | 18.9 |
| SK15 | 1 | 2614.7 | 94.0 | 123.0 | 238.0 | 13.3 |
|  | 2 | 2075.0 | 52.3 | 66.4 | 190.2 | 11.1 |
|  | 3 | 1456.5 | 53.0 | 42.4 | 169.2 | 8.8 |
|  | 4 | 1709.7 | 57.7 | 47.1 | 226.0 | 13.9 |
|  | 5 | 2416.4 | 93.7 | 107.6 | 213.5 | 12.3 |
|  | 6 | 1785.8 | 70.6 | 56.8 | 203.7 | 6.1 |
|  | 7 | 1164.5 | 55.6 | 43.7 | 93.3 | 11.9 |
| SK16 | 1 | 1258.1 | 27.6 | 46.6 | 118.6 | 8.8 |
|  | 2 | 1679.9 | 70.4 | 52.0 | 197.2 | 14.2 |
|  | 3 | 1286.9 | 39.1 | 42.6 | 150.6 | 6.7 |
|  | 4 | 2409.7 | 62.2 | 113.1 | 243.6 | 11.2 |
|  | 5 | 2711.1 | 77.0 | 96.1 | 246.4 | 19.5 |
|  | 6 | 1790.2 | 63.7 | 52.2 | 220.1 | 13.1 |
|  | 7 | 1494.8 | 48.8 | 40.6 | 176.5 | 11.2 |
| SK18 | 1 | 1311.0 | 52.0 | 32.9 | 142.0 | 8.0 |
|  | 2 | 2022.5 | 86.9 | 74.0 | 174.9 | 28.0 |
|  | 3 | 1414.0 | 54.0 | 52.9 | 147.5 | 16.4 |
|  | 4 | 1916.9 | 65.3 | 62.8 | 169.3 | 15.5 |
|  | 5 | 1695.9 | 87.2 | 87.5 | 103.3 | 14.8 |
|  | 6 | 2140.2 | 60.2 | 54.8 | 174.6 | 15.9 |
|  | 7 | 2895.9 | 111.4 | 111.5 | 249.4 | 23.8 |
| SK20 | 1 | 2357.6 | 49.6 | 89.9 | 280.2 | 19.7 |
|  | 2 | 3180.9 | 77.9 | 185.8 | 245.9 | 33.7 |
|  | 3 | 2315.5 | 60.4 | 91.3 | 266.2 | 19.6 |
|  | 4 | 2774.9 | 123.4 | 59.1 | 271.6 | 14.7 |
|  | 5 | 3168.9 | 97.1 | 137.8 | 240.2 | 23.9 |
|  | 6 | 1859.7 | 48.9 | 30.2 | 299.9 | 11.0 |
|  | 7 | 2264.2 | 44.9 | 64.7 | 326.3 | 31.3 |
| SK21 | 1 | 1140.1 | 54.4 | 44.1 | 100.3 | 8.4 |
|  | 2 | 1072.4 | 34.7 | 26.3 | 148.8 | 5.9 |
|  | 3 | 1151.6 | 44.4 | 33.4 | 129.9 | 7.2 |
|  | 4 | 1483.8 | 39.4 | 59.9 | 101.4 | 3.7 |
|  | 5 | 1105.0 | 44.9 | 36.0 | 124.3 | 6.0 |
|  | 6 | 1167.0 | 55.5 | 41.1 | 108.2 | 9.5 |
|  | 7 | 1356.0 | 33.0 | 48.3 | 169.7 | 3.4 |
| SK22 | 1 | 1203.6 | 46.3 | 31.2 | 158.1 | 7.7 |
|  | 2 | 2154.3 | 62.8 | 76.9 | 250.4 | 14.6 |
|  | 3 | 1374.2 | 35.8 | 51.3 | 165.1 | 8.4 |
|  | 4 | 1313.6 | 48.8 | 49.0 | 131.2 | 8.9 |
|  | 5 | 1777.2 | 74.2 | 65.0 | 156.3 | 12.5 |
|  | 6 | 1741.2 | 82.2 | 62.0 | 163.0 | 13.0 |
|  | 7 | 2593.1 | 146.0 | 116.6 | 179.0 | 5.0 |
| SK23 | 1 | 1905.9 | 59.8 | 76.2 | 178.4 | 18.1 |
|  | 2 | 2111.3 | 67.2 | 108.2 | 156.0 | 24.4 |
|  | 3 | 2006.5 | 65.8 | 104.6 | 162.0 | 24.2 |
|  | 4 | 2516.2 | 95.1 | 99.4 | 141.4 | 12.9 |
|  | 5 | 2565.9 | 123.9 | 94.8 | 119.1 | 24.8 |
|  | 6 | 2101.5 | 84.4 | 61.8 | 213.6 | 18.4 |
|  | 7 | 1262.8 | 38.5 | 27.0 | 184.4 | 9.1 |
| SK24 | 1 | 1938.7 | 49.5 | 63.7 | 241.5 | 11.7 |
|  | 2 | 1710.4 | 50.4 | 52.0 | 219.2 | 8.7 |
|  | 3 | 1746.9 | 56.9 | 78.8 | 154.8 | 9.0 |
|  | 4 | 1748.0 | 59.3 | 47.1 | 228.5 | 7.6 |
|  | 5 | 1688.3 | 68.4 | 50.1 | 198.8 | 8.9 |
|  | 6 | 2130.4 | 68.1 | 68.7 | 252.2 | 10.9 |
|  | 7 | 2141.8 | 68.9 | 59.0 | 280.9 | 10.7 |
| SK26 | 1 | 975.0 | 36.4 | 35.0 | 109.9 | 1.1 |
|  | 2 | 1300.6 | 45.6 | 66.5 | 105.3 | 8.8 |
|  | 3 | 2117.8 | 63.7 | 91.7 | 217.2 | 8.1 |
|  | 4 | 2758.4 | 69.0 | 82.8 | 237.4 | 9.4 |
|  | 5 | 1065.8 | 20.3 | 20.5 | 92.8 | 3.5 |
|  | 6 | 1096.4 | 35.1 | 51.2 | 102.5 | 7.5 |
|  | 7 | 1658.2 | 53.6 | 69.9 | 148.8 | 7.6 |
| SK27 | 1 | 1238.9 | 47.6 | 54.5 | 62.2 | 17.3 |
|  | 2 | 1790.2 | 75.6 | 69.1 | 141.3 | 20.4 |
|  | 3 | 1144.5 | 47.2 | 34.3 | 107.0 | 14.1 |
|  | 4 | 1058.1 | 37.4 | 38.4 | 119.1 | 7.1 |
|  | 5 | 509.6 | 13.0 | 24.9 | 30.2 | 0.2 |
|  | 6 | 1678.4 | 73.2 | 70.6 | 130.9 | 10.5 |
|  | 7 | 1396.7 | 53.2 | 64.4 | 93.4 | 8.1 |
| SK28 | 1 | 3653.2 | 116.9 | 154.6 | 269.2 | 25.7 |
|  | 2 | 1485.6 | 63.4 | 55.1 | 130.4 | 9.1 |
|  | 3 | 1567.0 | 53.8 | 51.0 | 189.0 | 8.9 |
|  | 4 | 1409.2 | 56.4 | 44.6 | 152.2 | 9.9 |
|  | 5 | 1776.3 | 74.4 | 64.7 | 162.9 | 13.2 |
|  | 6 | 2149.9 | 99.8 | 73.4 | 197.0 | 14.2 |
|  | 7 | 1612.4 | 47.9 | 54.5 | 185.0 | 11.1 |
| SK30 | 1 | 2052.3 | 69.6 | 77.9 | 210.3 | 19.6 |
|  | 2 | 1705.2 | 72.2 | 72.6 | 150.1 | 19.3 |
|  | 3 | 1736.5 | 51.5 | 22.6 | 271.1 | 18.9 |
|  | 4 | 2154.5 | 81.2 | 64.8 | 235.9 | 7.1 |
|  | 5 | 1361.2 | 55.1 | 53.6 | 136.5 | 11.5 |
|  | 6 | 2199.7 | 74.4 | 105.0 | 182.6 | 15.3 |
|  | 7 | 2140.3 | 66.6 | 95.9 | 191.4 | 9.7 |
| SK32 | 1 | 1985.0 | 71.8 | 34.8 | 244.5 | 22.2 |
|  | 2 | 2290.4 | 79.0 | 53.4 | 286.3 | 22.7 |
|  | 3 | 1911.4 | 74.6 | 52.4 | 234.4 | 20.0 |
|  | 4 | 2411.8 | 81.7 | 84.9 | 258.1 | 25.8 |
|  | 5 | 1835.4 | 64.5 | 56.4 | 221.7 | 11.0 |
|  | 6 | 3002.2 | 97.8 | 145.0 | 283.4 | 18.3 |
|  | 7 | 2534.8 | 74.0 | 92.3 | 271.8 | 23.2 |
| Term2 | 1 | 1796.2 | 68.4 | 45.1 | 238.4 | 11.7 |
|  | 2 | 1659.6 | 54.7 | 26.7 | 252.5 | 13.1 |
|  | 3 | 1533.6 | 57.4 | 20.2 | 235.5 | 16.7 |
|  | 4 | 1741.5 | 54.4 | 38.7 | 199.7 | 11.1 |
|  | 5 | 1616.1 | 49.9 | 23.6 | 251.7 | 12.6 |
|  | 6 | 1759.9 | 48.7 | 56.3 | 200.6 | 9.8 |
|  | 7 | 2004.5 | 55.7 | 63.9 | 256.4 | 9.5 |
| Term3 | Tofu | 1999.2 | 60.7 | 33.1 | 307.8 | 18.7 |
| Term3 | Meat | 2387.6 | 76.9 | 64.3 | 315.3 | 18.8 |

**Table S3. Correlation between stability of the metabolome, stability of the microbiome, and α-diversity as related to Fig. S5.**

| Pair 1 | Pair 2 | Spearman's correlation coefficient | q-Value |
| --- | --- | --- | --- |
| Faith PD | Stability of metabolome | –0.693 | 0.038 |
| Chao1 index | Stability of metabolome | –0.586 | 0.102 |
| Stability of food choice | Stability of metabolome | –0.554 | 0.122 |
| Shannon index | Stability of food choice | 0.356 | 0.173 |
| Chao1 index | Stability of food choice | 0.354 | 0.173 |
| Shannon index | Stability of metabolome | –0.486 | 0.173 |
| Shannon index | Stability of weighted UniFrac | –0.471 | 0.173 |
| Faith PD | Stability of food choice | 0.240 | 0.402 |
| Stability of metabolome | Stability of weighted UniFrac | 0.329 | 0.402 |
| Stability of metabolome | Stability of unweighted UniFrac | 0.275 | 0.480 |
| Chao1 index | Stability of weighted UniFrac | –0.214 | 0.619 |
| Shannon index | Stability of unweighted UniFrac | 0.182 | 0.676 |
| Stability of food choice | Stability of unweighted UniFrac | –0.164 | 0.689 |
| Stability of food choice | Stability of weighted UniFrac | 0.132 | 0.745 |
| Chao1 index | Stability of unweighted UniFrac | 0.111 | 0.768 |
| Faith PD | Stability of unweighted UniFrac | 0.086 | 0.801 |
| Faith PD | Stability of weighted UniFrac | 0.004 | 0.995 |

Stability score were based on Bray-Curtis distance of metabolome and food choice profiles and UniFrac distance (weighted and unweighted) of microbiome profiles within each individual during term 1. α-diversity of microbiome profiles were evaluated using Faith PD, Chao1 and Shannon index.

**Table S4. Correlation between food choices, genus-level microbiome data, and metabolites**

| Food choice | Genus/metabolites | Spearman’s rank correlation coefficients | FDR | Subject |
| --- | --- | --- | --- | --- |
| Teas | Azelate | -0.82 | 0.04 | SK14 |
| Teas | Azelate | -0.74 | 0.12 | SK20 |
| Other seasonings | g_LachnospiraceaeFCS020group | -0.71 | 0.16 | SK24 |
| Other seasonings | g_LachnospiraceaeFCS020group | 0.68 | 0.19 | SK20 |
| Bread | Arg | 0.86 | 0.11 | SK10 |
| Bread | Arg | 0.75 | 0.13 | SK14 |
| Other wheat products | Arg | -0.86 | 0.11 | SK10 |
| Other wheat products | Arg | 0.72 | 0.15 | SK24 |
| Rice | Azelate | 0.83 | 0.04 | SK20 |
| Rice | Azelate | 0.78 | 0.08 | SK14 |
| Other wheat products | Dihydrouracil | 0.76 | 0.10 | SK20 |
| Other wheat products | Dihydrouracil | -0.90 | 0.17 | SK32 |
| Other wheat products | g_Roseburia | 0.84 | 0.04 | SK24 |
| Other wheat products | g_Roseburia | 0.74 | 0.15 | SK14 |
| Flour | Inosine | 0.80 | 0.06 | SK14 |
| Flour | Inosine | -0.72 | 0.15 | SK24 |
| Other wheat products | Lactate | -0.93 | 0.07 | SK32 |
| Other wheat products | Lactate | -0.68 | 0.19 | SK24 |
| Other wheat products | Met | -0.80 | 0.07 | SK20 |
| Other wheat products | Met | -0.88 | 0.08 | SK10 |
| Eggs | Imidazole-4-acetate | -0.98 | 0.00 | SK32 |
| Eggs | Imidazole-4-acetate | -0.89 | 0.05 | SK10 |
| Other raw fishes | Purineriboside | 0.98 | 0.00 | SK01 |
| Other raw fishes | Purineriboside | 0.86 | 0.02 | SK14 |
| Banana | Citraconate | 0.73 | 0.13 | SK20 |
| Banana | Citraconate | 0.85 | 0.17 | SK04 |
| Banana | Dodecanedioate | 0.74 | 0.12 | SK24 |
| Banana | Dodecanedioate | 0.68 | 0.19 | SK20 |
| Banana | g_Blautia | -0.73 | 0.13 | SK20 |
| Banana | g_Blautia | -0.72 | 0.17 | SK14 |
| Banana | g_Streptococcus | -0.72 | 0.15 | SK24 |
| Banana | g_Streptococcus | -0.86 | 0.17 | SK04 |
| Banana | N1,N12-Diacetylspermine | -0.77 | 0.09 | SK24 |
| Banana | N1,N12-Diacetylspermine | -0.84 | 0.16 | SK10 |
| Banana | Pimelate | 0.77 | 0.10 | SK24 |
| Banana | Pimelate | 0.84 | 0.16 | SK10 |
| Banana | Pimelate | 0.85 | 0.17 | SK04 |
| Banana | SDMA | -0.72 | 0.15 | SK24 |
| Banana | SDMA | -0.85 | 0.17 | SK04 |
| Hams and sausages | Phe | 0.85 | 0.18 | SK04 |
| Hams and sausages | Phe | 0.68 | 0.20 | SK24 |
| Spices and others | Pimelate | 0.86 | 0.03 | SK24 |
| Spices and others | Pimelate | 0.88 | 0.12 | SK04 |
| Fermented milk and lactic acid bacteria beverages | Phenethylamine | 0.85 | 0.03 | SK20 |
| Fermented milk and lactic acid bacteria beverages | Phenethylamine | 0.73 | 0.15 | SK14 |
| Vegetable fats and oils | Glu | -0.72 | 0.15 | SK20 |
| Vegetable fats and oils | Glu | -0.74 | 0.15 | SK14 |
| Soy sauces | N1,N12-Diacetylspermine | -0.88 | 0.08 | SK10 |
| Soy sauces | N1,N12-Diacetylspermine | -0.71 | 0.16 | SK24 |
| Sugar and sweetener | Sebacate | 0.77 | 0.09 | SK24 |
| Sugar and sweetener | Sebacate | -0.92 | 0.12 | SK32 |
| Other potatoes/Other potatoes products | Glycocholate | 0.76 | 0.11 | SK14 |
| Other potatoes/Other potatoes products | Glycocholate | 0.85 | 0.18 | SK04 |
| Other potatoes/Other potatoes products | Pimelate | -0.94 | 0.05 | SK32 |
| Other potatoes/Other potatoes products | Pimelate | 0.84 | 0.19 | SK04 |
| Age (Fried Tofu) | g_Anaerostipes | 0.87 | 0.12 | SK01 |
| Age (Fried Tofu) | g_Anaerostipes | 0.86 | 0.17 | SK04 |
| Tofu | Met | -0.84 | 0.16 | SK10 |
| Tofu | Met | -0.89 | 0.17 | SK32 |
| Tofu | N1,N12-Diacetylspermine | -0.73 | 0.13 | SK24 |
| Tofu | N1,N12-Diacetylspermine | -0.84 | 0.16 | SK10 |
| Tofu | Phe | -0.84 | 0.16 | SK10 |
| Tofu | Phe | -0.69 | 0.18 | SK24 |
| Tofu | Pimelate | 0.76 | 0.11 | SK24 |
| Tofu | Pimelate | 0.84 | 0.16 | SK10 |
| Tofu | Pimelate | 0.85 | 0.17 | SK04 |
| Tofu | Prolinebetaine | 0.82 | 0.06 | SK24 |
| Tofu | Prolinebetaine | -0.75 | 0.13 | SK14 |
| Other Green and yellow vegetables | 2AB | -0.76 | 0.10 | SK20 |
| Other Green and yellow vegetables | 2AB | -0.70 | 0.17 | SK24 |
| Cabbage | Cholate | -0.89 | 0.06 | SK01 |
| Cabbage | Cholate | 0.76 | 0.10 | SK24 |
| Onion | g_Faecalibacterium | 0.76 | 0.11 | SK24 |
| Onion | g_Faecalibacterium | 0.74 | 0.12 | SK20 |
| Onion | g_Subdoligranulum | 0.76 | 0.11 | SK24 |
| Onion | g_Subdoligranulum | -0.75 | 0.12 | SK20 |
| Spinach | Glu | 0.79 | 0.07 | SK20 |
| Spinach | Glu | -0.78 | 0.09 | SK14 |
| Other Green and yellow vegetables | Gly-Gly | -0.74 | 0.12 | SK24 |
| Other Green and yellow vegetables | Gly-Gly | -0.72 | 0.15 | SK20 |
| Spinach | Glycocholate | 0.91 | 0.06 | SK04 |
| Spinach | Glycocholate | 0.77 | 0.10 | SK14 |
| Other Green and yellow vegetables | His | -0.83 | 0.05 | SK20 |
| Other Green and yellow vegetables | His | -0.74 | 0.12 | SK24 |
| Daikon | Isethionate | 0.82 | 0.04 | SK14 |
| Daikon | Isethionate | -0.75 | 0.11 | SK24 |
| Other Green and yellow vegetables | *N*-Acetylglutamate | 0.83 | 0.04 | SK20 |
| Other Green and yellow vegetables | *N*-Acetylglutamate | 0.75 | 0.11 | SK24 |
| Carrot | *N-*Acetylornithine | -0.93 | 0.02 | SK04 |
| Carrot | *N*-Acetylornithine | -0.70 | 0.17 | SK24 |
| Onion | N1,N12-Diacetylspermine | -0.83 | 0.05 | SK24 |
| Onion | N1,N12-Diacetylspermine | -0.85 | 0.14 | SK10 |
| Other Green and yellow vegetables | o-Acetylcarnitine | -0.76 | 0.10 | SK20 |
| Other Green and yellow vegetables | o-Acetylcarnitine | -0.72 | 0.14 | SK24 |
| Onion | Pimelate | 0.88 | 0.07 | SK10 |
| Onion | Pimelate | 0.75 | 0.11 | SK24 |
| Other vegetables | Pimelate | -0.97 | 0.01 | SK32 |
| Other vegetables | Pimelate | 0.89 | 0.09 | SK04 |
| Daikon | SDMA | -0.74 | 0.12 | SK24 |
| Daikon | SDMA | -0.87 | 0.13 | SK04 |
| Spinach | SDMA | -0.90 | 0.07 | SK04 |
| Spinach | SDMA | -0.71 | 0.16 | SK24 |
